# 16S rRNA gene profiling and genome reconstruction reveal community metabolic interactions and prebiotic potential of medicinal herbs used in neurodegenerative disease and as nootropics

**DOI:** 10.1371/journal.pone.0213869

**Published:** 2019-03-19

**Authors:** Christine Tara Peterson, Vandana Sharma, Stanislav N. Iablokov, Levent Albayrak, Kamil Khanipov, Sasha Uchitel, Deepak Chopra, Paul J. Mills, Yuriy Fofanov, Dmitry A. Rodionov, Scott N. Peterson

**Affiliations:** 1 UC San Diego, School of Medicine, Center of Excellence for Research and Training in Integrative Health, Department of Family Medicine and Public Health, La Jolla, California, United States of America; 2 Sanford Burnham Prebys Medical Discovery Institute, Bioinformatics and Structural Biology Program, La Jolla, California, United States of America; 3 Institute for Information Transmission Problems, Russian Academy of Sciences, Moscow, Russia; 4 P.G. Demidov Yaroslavl State University, Yaroslavl, Russia; 5 Department of Pharmacology and Toxicology, Sealy Center for Structural Biology, University of Texas Medical Branch, Galveston, Texas, United States of America; 6 Washington University, Department of Biology, St. Louis, Missouri, United States of America; 7 Chopra Foundation, Department of Ayurveda and Yoga Research, Carlsbad, California, United States of America; 8 Sanford Burnham Prebys Medical Discovery Institute, Tumor Microenvironment and Cancer Immunology Program, La Jolla, California, United States of America; University of Illinois, UNITED STATES

## Abstract

The prebiotic potential of nervine herbal medicines has been scarcely studied. We therefore used anaerobic human fecal cultivation to investigate whether medicinal herbs commonly used as treatment in neurological health and disease in Ayurveda and other traditional systems of medicine modulate gut microbiota. Profiling of fecal cultures supplemented with either *Kapikacchu*, *Gotu Kola*, *Bacopa/Brahmi*, *Shankhapushpi*, *Boswellia/Frankincense*, *Jatamansi*, *Bhringaraj*, *Guduchi*, *Ashwagandha* or *Shatavari* by 16S rRNA sequencing revealed profound changes in diverse taxa. Principal coordinate analysis highlights that each herb drives the formation of unique microbial communities predicted to display unique metabolic potential. The relative abundance of approximately one-third of the 243 enumerated species was altered by all herbs. Additional species were impacted in an herb-specific manner. In this study, we combine genome reconstruction of sugar utilization and short chain fatty acid (SCFA) pathways encoded in the genomes of 216 profiled taxa with monosaccharide composition analysis of each medicinal herb by quantitative mass spectrometry to enhance the interpretation of resulting microbial communities and discern potential drivers of microbiota restructuring. Collectively, our results indicate that gut microbiota engage in both protein and glycan catabolism, providing amino acid and sugar substrates that are consumed by fermentative species. We identified taxa that are efficient amino acid fermenters and those capable of both amino acid and sugar fermentation. Herb-induced microbial communities are predicted to alter the relative abundance of taxa encoding SCFA (butyrate and propionate) pathways. Co-occurrence network analyses identified a large number of taxa pairs in medicinal herb cultures. Some of these pairs displayed related culture growth relationships in replicate cultures highlighting potential functional interactions among medicinal herb-induced taxa.

## Introduction

Millions of individuals are adversely affected by neurodegenerative disease worldwide [1]. Global health improvements have increased human lifespan, which further exacerbates this disease burden. Neurodegenerative diseases, such as Parkinson’s Disease (PD) and Alzheimer’s Disease (AD), represent a heterogenous group of disorders that promote deterioration of the central and/or peripheral nervous systems and affect an estimated 1% and 8% of the population, respectively [2]. Nootropics, which are drugs, supplements or herbal medicines that exert action on the nervous system for increased mental performance, are increasingly used by both healthy individuals and individuals with neurodegenerative diseases [3–5]. A large Global Drug Survey of over 100,000 participants recently reported that 30% of respondents had taken nootropics for cognitive enhancement and that nearly half of the users had obtained the cognition enhancers through friends [6]. Thus, a burgeoning need exists for the evaluation of the efficacy of these products and the investigation of mechanisms of action through which medicinal herbs impinge on the progression of neurodegenerative diseases and to safely support cognition in healthy individuals.

Recent studies suggest that altered gut microbiota and its metabolites are associated with neurodegenerative diseases such as PD and AD; however, the causal relationships with human microbiota have yet to be established [7]. In AD patients, Bacteroidetes was decreased, whereas Actinobacteria was slightly more abundant compared to gender-matched controls. Reduced relative abundance of other butyrate producers from the Lachnospiraceae family such as Coprococcus, Faecalibacterium and Roseburia species has been observed in PD stool compared to healthy controls [8–10]. Butyrate, a short chain fatty acid (SCFA), displays pleiotropic effects on host physiology which can inhibit histone deacetylase, proinflammatory cytokines, promote improved gut barrier function, induce Tregs, and function as a gut-brain axis signaling molecule [11, 12]. SCFAs also attenuate neuroimmune mechanisms, neuroinflammatory processes driving inflammaging, and the integrity of the blood-brain-barrier (BBB) [13, 14]. SCFAs such as butyrate are reduced in PD stool compared to age-matched controls and is a relevant clinical consideration in patients given the anti-inflammatory and neuroprotective effects of these bacterial fermentation products [15].

Herbal medicines used for neurological health and disease were the subject of the current study (Table 1). The traditional system of medicine in India, namely Ayurveda, emphasizes gastrointestinal health and disease prevention and commonly uses these medicinal herbs for neurological health and disease. These nervine herbal medicines contain compounds that cross the BBB [16] and likely interact with gut microbiota to induce local and systemic effects including alterations in the gut-brain axis. Use of these herbal medicines is widespread for support in neurodegenerative diseases such as AD and PD as well as in healthy populations such as medical students for nootropic effects [16–19].

**Table 1:** Nervine herbal medicines examined in the current study.

Species	Common Name	Family
*Bacopa monnieri*	brahmi or waterhyssop	Plantaginaceae
*Evolvulus alsinoides*	shankhapushpi	Convolvulaceae
*Centella asiatica*	gotu kola or pennywort	Apiaceae
*Nardostachys jatamansi*	Jatamansi	Valerianaceae
*Boswellia serrata*	Frankincense	Burseraceae
*Eclipta alba*	bhringaraj or false daisy	Asteraceae
*Mucuna pruriens*	kapikacchu or velvet bean	Fabaceae
*Withania somnifera*	Ashwagandha	Solanaceae
*Asparagus racemosus*	Shatavari	Asparagaceae
*Tinospora cordifolia*	Guduchi	Menispermaceae

Selected common names and family information are shown. Note that in some regions in India, brahmi may refer to *C*. *asiatica*. In addition, shankhapushpi may refer to any of 4 nervine species to include *Convulvulus pluricaulis* (Convulvulaceae), *Evolvulus alsinoides* (Convulvulaceae), *Clitoria ternatea* (Papilionaceae) and *Canscora decussata* (Gentianaceae); the commonly used *E*. *alsinoides* was examined here.

Our recent work has established the prebiotic potential of medicinal herbs [20, 21]. The most commonly cited yet debated definition of prebiotics refers to dietary carbohydrates selectively fermented by gut microbiota that modulate the composition of microbiota to confer health benefits to the host [22, 23]. Glycan prebiotics are catabolized by microbes such as *Bifidobacterium* spp. and *Bacteroides* spp. in the colon. Sugars liberated by these species and others are then fermented by a large repertoire of saccharolytic species. The end products of microbial carbohydrate and amino acid metabolism include SCFAs. The prebiotic potential of herbal medicines to maintain immune homeostasis, reduce inflammation, improve colonic barrier function, promote protection from opportunistic pathogens, and modulate the gut-brain axis warrants additional basic research and clinical investigation.

Unlike traditional glycan prebiotics, the medicinal herbs studied here provide both complex carbohydrate and protein substrates. An important consequence of medicinal herb-driven prebiotic effects is the alterations in bacterial community metabolism, which is expected to result in the generation of unique metabolites that may contribute to therapeutic efficacy. Addressing these possibilities will require human intervention studies.

In the current investigation, anaerobic human fecal cultivation was used to investigate the extent to which 10 nervine herbal medicines commonly used in both neurodegenerative disease and as nootropics alter the growth and abundance of gut bacterial species. It is either currently unknown or scarcely little is known about the gut microbiota in the context of each herbal medicine. It is uncertain if and to what extent gut microbiota or their metabolites mediate the effects of these medicinal herbs. Mass spectrometry was implemented to determine monosaccharide profiles of the 10 medicinal herbs since this basic information was largely incomplete in the scientific literature. Genome reconstruction was applied to determine sugar utilization, SCFA production and glycosyl hydrolase potential in the context of sugar profiles. Co-occurrence network analysis was implemented to identify community interactions and cross-feeding relationships. Thus, the authors hypothesized that the substrates present in herbal medicines may be potent drivers to alter the gut microbiota composition thereby redirecting community metabolism.

## Methods

### Study participants and sample collection

Twelve healthy, English-speaking women and men aged 30–60 years that had previously adhered to a vegetarian or vegan diet for >1 year were recruited to donate a single stool sample. This study was carried out in accordance with the recommendations of Sanford Burnham Prebys Medical Discovery Institute Institutional Review Board and guidelines with written informed consent from all subjects. All subjects gave written informed consent in accordance with the Declaration of Helsinki. The protocol was approved by the Sanford Burnham Prebys Medical Discovery Institute Institutional Review Board (IRB-2014-020). Participants ate their normal diets and donated a morning fecal sample in stool hats (Fisher Scientific). The fecal samples were transferred to conical tubes and stored at -80°C until further processing.

### Nervine medicinal herbs examined in the current microbiome study

We examined 10 medicinal herbs in this study (Table 1) sourced from Banyan Botanicals (Albuquerque, NM), with the exception of Jatamansi, which was sourced from AyurOrganics (Victoria, Australia).

### Anaerobic fecal cultures

Equal volumes of stool collected from 12 healthy vegetarian participants were pooled into a single sample and used to inoculate (approximately 10^6^ cells) a chemically defined medium (CDM) or CDM supplemented with either 1% herb or 1% glucose in Hungate tubes. Anaerobic cultures (9% H_2_, 81% N_2_) were grown statically for 3–4 days at 37°C as technical replicates (n = 6) and grown to approximate saturation. CDM contains 50 mM HEPES, 2.2 mM KH_2_PO_4_, 10 mM Na_2_HPO_4_, 60 mM NaHCO_3_, 4 mM of each amino acid, except leucine (15 mM), 10 mL ATCC, Trace Mineral Supplement. CDM contained nucleoside bases (100 mg/L), inosine, xanthine, adenine, guanine, cytosine, thymidine and uracil (400 mg/L). CDM contained choline (100 mg/L), ascorbic acid (500 mg/L), lipoic acid (2 mg/L), hemin (1.2 mg/L) and myo-inositol (400 mg/L). Resazurin (1 mg/L) was added to visually monitor dissolved oxygen. The pH of the media was adjusted to 7.4. The 2X CDM and medicinal herbs (powder) in sterile water (2%) were separately reduced in an anaerobic chamber (Coy Labs) for 3 days.

### Microbial DNA Isolation

Genomic DNA was isolated from cultures as well as the fecal inoculum using the procedures of the QiaAmp DNA stool kit (Qiagen) with a modification that included an additional step of bead beating using the Thermo FastPrep instrument (MP Bio) to ensure uniform lysis of bacterial cells. DNA was purified with QIAquick (Qiagen) purification kit columns. DNA integrity was analyzed by spectrophotometry and visualized by gel electrophoresis. Quantitative PCR was used to allow equivalent amounts of each amplicon generated in each sample to be pooled for library construction.

### 16S rRNA sequence analysis

Multiplexed 16S rRNA libraries were prepared using standard 16S metagenomic sequencing library protocols from Illumina, which uses V3-V4 region of 16S rRNA for target amplification. We performed paired end reads (250 bp) sequencing to generate ~200,000 sequences/sample using the Illumina MiSeq. Subsequent analysis was done in CLC Microbial Genomics Module 2.5 (Qiagen) and R [24]. Paired end reads were merged (mismatch cost– 2, minimum score– 8, gap cost– 3, maximum unaligned end mismatches– 0) and trimmed to the same length. Additional quality filter steps were applied to exclude short reads, sequences with poor quality scores, and chimeras. To ensure comparable high coverage in all samples, we excluded samples producing <35,000 high quality reads. We did not use OTU-based enumeration of taxa due to the over-merging that occurs. Instead each unique 16S rRNA sequence was subjected to BLAST using the NCBI 16S rRNA database (Bacteria and Archaea) to identify best matches to taxa at the genus and species levels based on % identity.

### Statistical analyses

Differential abundance in the microbiome across treatment groups was evaluated using the Analysis of Composition of Microbiomes (ANCOM) methodology [25] with a False Discovery Rate adjusted p-value of 0.05 threshold for significance. The ANCOM procedure compares the relative abundance of a taxon between two ecosystems by computing Aitchison’s log-ratio of abundance of each taxon relative to the abundance of all other taxa one at a time. The significance of each test is determined using the Benjamini-Hochberg procedure that controls for FDR at 0.05. To account for taxa absent in sample groups, an arbitrary pseudocount of 1 is used. Weighted UniFrac distances and beta-diversity (Bray-Curtis) values were calculated using the Qiagen CLC Genomics Module, which provides visualization of the PCoA plot.

### Mass spectrometry analysis of nervine herbal medicines

All samples (1% w/v) were hydrolyzed with 2M TFA at 100°C for 4 hrs. Samples were dried to completion under nitrogen and reconstituted in MilliQ water. Samples were filtered through pre-washed Costar Spin-X filters (0.45μm, nylon). An aliquot of each sample (1%) was injected onto HPLC. Monosaccharide analysis was performed using Dionex CarboPac™ PA1 column (4X250 mm) with PA1 guard column (4x50 mm) at a flow rate of 1 ml/min. Monosaccharide detection was with pulsed amperometric detection with gold electrode. Elution gradients were as follows: 0–20 min, 19 mM NaOH, 20–50 min, 0 mM-212.5 mM NaOAc gradient with 19 mM NaOH, 50–65 min, 212.5 mM NaOAc with 19 mM NaOH, 65–68 min, 212.5 mM–0 mM NAOAc with 19 mM NaOH, 68–85 min, 19 mM NaOH. Standards included: fucose, rhamnose, arabinose, galactose, glucose, mannose, xylose, fructose, ribose, galacturonic acid and glucuronic acid. The monosaccharides were assigned based on the retention time and quantified using Chromeleon™ 6.8 chromatography data system software.

### Genome reconstruction of sugar metabolism and SCFA pathways

To predict metabolic capabilities of microbial taxa identified by 16S analysis, we performed genomics-based reconstruction of ten metabolic subsystems including eight subsystems involved in sugar uptake and utilization and two subsystems for SCFA synthesis. We used a subsystems-based approach implemented in the SEED genomic platform [26] to capture, analyze and extend pathways, enzymes, and transporters involved in sugar and SCFA metabolism in microbial genomes. This approach is based on functional gene annotation and prediction using three comparative genomic techniques: (i) homology-based methods; (ii) genome context analysis; (iii) co-regulation by the same regulon [27]. These context-based techniques were used to disambiguate paralogs with related but distinct functions (most notably transporters) and fill-in gaps (“missing genes”) in the inferred biochemical pathways.

The reference set of 2,228 genomes representing ~700 microbial species from human gut were from the PATRIC genomic database [28]. The metabolic subsystems were developed based on previously published genomic studies of sugar metabolism in various bacterial taxa [28–32] and the studies of phylogenetic distribution of bacterial pathways for production of butyrate [33] and propionate [34]. Each reference genome in each analyzed sugar subsystem was assigned a binary (“1” or “0”) phenotype reflecting the presence/absence of a complete sugar utilization pathway including a sugar-specific uptake transporter (S1 Fig). For SCFA subsystems, the assigned binary phenotypes reflect the presence/absence of at least one functional pathway variant, as both propionate and butyrate subsystems include four different pathway variants (S2 Fig). The obtained binary phenotype matrix (BPM) for reference genomes was used to calculate a community phenotype matrix (CPM) for all mapped taxa obtained from 16S analysis by averaging the respective CPM values (S2 and S5 Tables). Community phenotype index (CPI) for each sample was calculated as the sum of respective CPM values of each taxa multiplied by their relative abundances. CPI gives a probabilistic estimate of a fraction of the community possessing a specific metabolic pathway (0–100%).

### Co-occurrence analysis

Complex pairwise interactions (such as co-presence, co-exclusion, and one-way dependence) among organisms were screened using mutual information [35] with software developed in-house for the purpose of this project in C++. Mutual information, similar to C-score [36], operates on presence/absence data. Individual presence/absence relative abundance thresholds were identified for each pairwise interaction by maximizing mutual information across a threshold plane formed by relative abundance values of interacting organisms. The bootstrapping approach used to estimate the statistical significance of the patterns allows the removal of low fidelity patterns that may appear simply by chance. All combinations of taxa pairs were used for screening, only pairs which perform above a predefined significance value were inspected. Bootstrapping was performed by randomly reshuffling the presence/absence vectors for each organism pair 1000 times. All patterns analyzed had p values <0.001.

## Results

### Nervine herbal medicines alter fecal microbiota

The fecal inoculums used for cultivation were derived from 12 healthy vegetarian donors and subsequently pooled in equal amounts into one pooled sample to increase species diversity. In total, 16S rRNA analysis revealed an estimated 317 distinct taxa, and we further analyzed 243 taxa that were observed at an average relative abundance >0.01% in at least one culture condition. Variability in technical replicate cultures negatively impacted the statistical significance of some taxa that are strongly altered in some but not all replicates (S1 Table).

**Fig 1:**
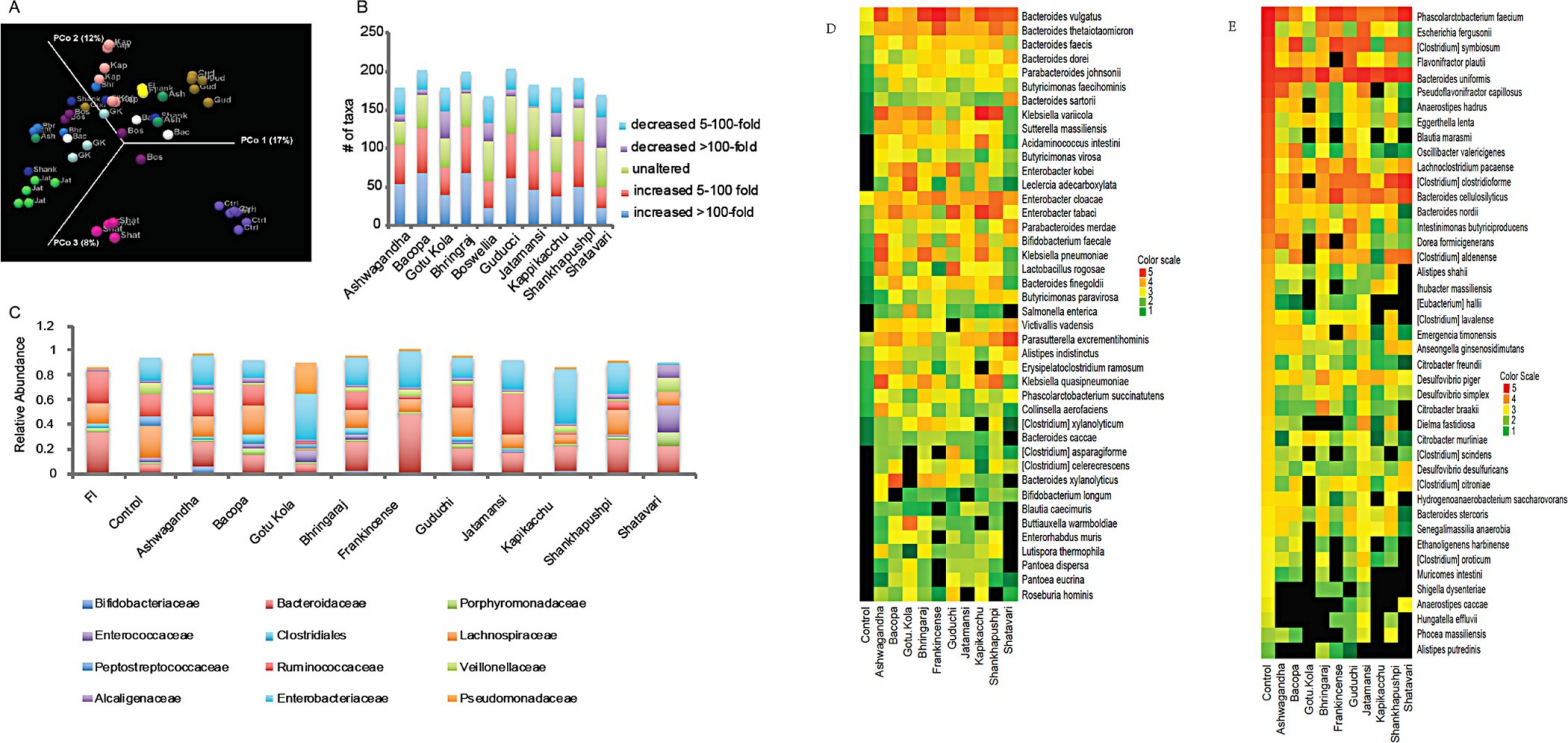
A. PCoA of nervine herbs. Bray-Curtis PCoA β-diversity plots of communities observed in Ctrl = control, Shat = Shatavari, Jat = Jatamansi, GK = Gotu Kola, Ash = Ashwagandha, Bos = Boswellia = Frankincense, Bac = Bacopa, Shank = Shankhapushpi, Gud = Guduchi, Kap = Kapikacchu, Bhr = Bhringaraj cultures and the uncultured FI = fecal inoculum. B. Modulatory capacity of nervine medicinal herbs. Average fold change of taxa comparing herb-supplemented to control cultures. Zeros were replaced with e-^6^ to permit minimum fold-change values to be calculated. C. Medicinal herbs induce distinct microbial communities. Average relative abundance of bacterial families generated in herb-supplemented cultures. D. Taxa displaying high herb responsiveness. Taxa displaying increased average abundance >5-fold compared to controls in 8 or more herb-supplemented cultures. Relative abundance values were multiplied by 1X10^6^ to convert all values to >1. These values were log_10_ transformed and depicted in the heatmap. E. Taxa displaying low herb responsiveness. Taxa displaying decreased average abundance >5-fold compared to controls in all herb-supplemented cultures. Relative abundance values were multiplied by 1X10^6^ to convert all values to >1. These values were log_10_ transformed and depicted in the heatmap.

PERMANOVA analysis (Bray Curtis) was used (CLC Microbial Genomics Module) and visualized with Principal Coordinate Analysis (PCoA) to identify the significance of the differences in community composition. All herb cultures were significantly different than control cultures (p<0.01–0.002)., visualized by pronounced shifts of microbial communities compared to non-supplemented control cultures (Fig 1A). Each herb-supplemented culture was significantly different than all others (p<0.04–0.002), with three exceptions. Communities generated in Ashwagandha, Bacopa and Gotu Kola were not significantly different with respect to each other. Shatavari, Jatamansi, Kapikacchu and Guduchi drive unique communities whereas the remaining medicinal herbs generate diverse communities that are more inter-related. PCoA analysis also highlights within-replicate culture variation. This variation is based on selection for distinct communities, rather than poor biological reproducibility of individual taxa. We calculated the average fold-change of taxa for each herb-supplemented culture relative to control cultures. The greatest overall modulatory capacity was observed in cultures supplemented with Bacopa, Guduchi, Bhringaraj, Ashwagandha and Shankhapushpi (Fig 1B). Each medicinal herb generated communities displaying unique dominance patterns of bacterial families (Fig 1C). Compared to control cultures lacking any carbohydrate energy source, Ashwagandha, Bhringaraj, Guduchi and Kapikacchu stimulated the growth of Bifidobacteriaceae and Bacteroidaceae, whereas Gotu kola communities were dominated by Enterobacteriaceae and Pseudomonadaceae. Frankincense strongly increased the relative abundance of Bacteroidaceae, and Jatamansi stimulated the expansion of Ruminococcaceae. Shatavari selected for a relatively balanced representation of several families including: Bacteroidaceae, Porphorymonadaceae, Enterococcaceae, Lachnospiraceae, Veillonellaceae and Alcaligenaceae. These results emphasize the strong and unique selective properties of herbal medicine substrates and the complexity of the induced communities involved in the coordinated catabolism and metabolism of each herb.

At the species level, 136 (56%) of observed taxa are altered by more than 100-fold by at least 1 medicinal herb. The relative abundance of 14 taxa (5.3%) was induced above control cultures by all 10 medicinal herbs including: *Acidaminococcus intestini*, 5 *Bacteroides* spp., including *B*. *thetaiotaomicron*, 2 *Butyricimonas* spp., *Enterobacter kobei*, *Klebsiella varicola*, *Leclercia adecarboxylata*, *Parabacteroides johnsonii*, *Sutterella massiliensis* and *Clostridium colinum*. In addition, 45 taxa (18%) displayed elevated relative abundance in response to 8 or more herbal medicines relative to control cultures (Fig 1D). The taxa displaying high herb-responsiveness are diverse but enriched for *Bacteroides* spp. The relative abundance of 45 taxa (18%) were never increased by any herb (Fig 1E). Nearly all of the non-responsive taxa were observed at high abundance (>0.1%) in control cultures, suggesting that these taxa may participate in herb metabolism as their relative abundance remains high in many medicinal herb-supplemented cultures.

### Sugar composition of nervine medicinal herbs

**Fig 2:**
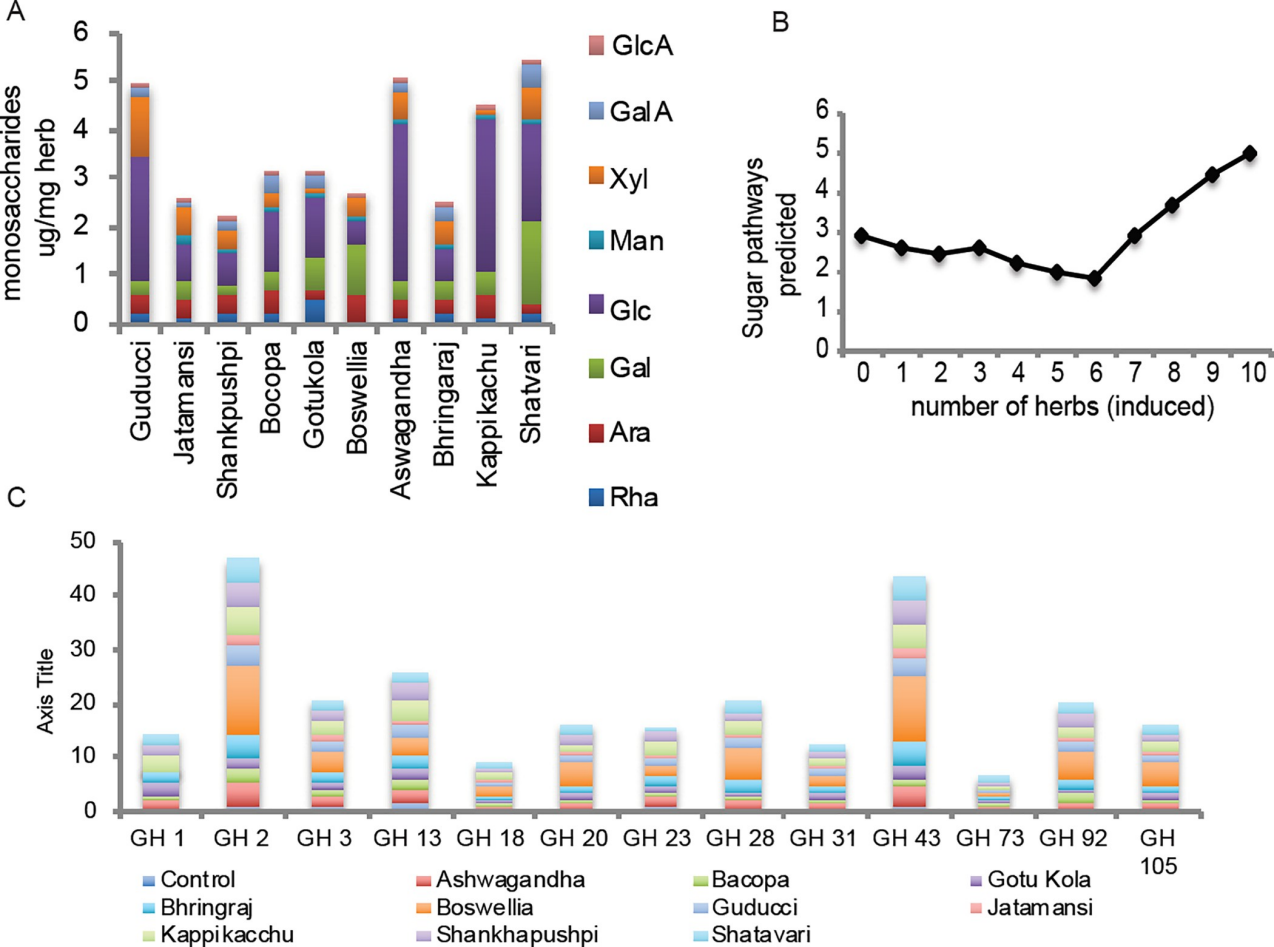
A. Monosaccharide composition of medicinal herbs. Proportions of monosaccharides detected in medicinal herbs: glucuronic acid, galacturonic acid, xylose, mannose glucose, galactose, arabinose and rhamnose. Fructose, fucose, ribose or glucosamine were not detected. B. Sugar utilization potential and herb-responsiveness. The average sugar utilization pathways for all taxa responding to 10, 9, 8 to 0 herbs. C. Modulation of glycosyl hydrolase representation. The relative abundance of taxa was multiplied by the number of genes in each GH family and summed for each herb.

To enhance our ability to interpret the microbiota profiles induced by each herbal medicine, we used quantitative HPAEC-PAD mass spectrometry to characterize the monosaccharide composition of each herb. Given the diverse taxonomy of the plants from which the studied medicinal herbs are derived, it was surprising that the monosaccharide profiles of all nervine herbal medicines contain a similar distribution of sugars (Fig 2A). We did not detect fructose, fucose, ribose or glucosamine in any herbal medicines. Additional sugar standards were not tested. With the exception of Guduchi, which lacked mannose, the remaining sugars are present in all of the medicinal herbs analyzed in varying abundance. Shatavari, Ashwagandha, Guduchi and Kapikacchu had the highest sugar content with glucose as the dominant monosaccharide. Frankincense and Shatavari displayed elevated proportions of galactose, whereas Guduchi possessed higher proportions of xylose.

### Sugar utilization capabilities of nervine medicinal herb-supplemented communities

Using a genomic approach, we performed metabolic reconstruction and obtained binary sugar utilization phenotypes for 8 monosaccharides detected in the medicinal herbs. A schematic of the sugar uptake transporters and catabolic enzymes involved in the reconstructed utilization pathways is provided in S1 Fig. The obtained community phenotype matrix (CPM) contains the sugar utilization capacity values for 216 taxa observed in medicinal herb-supplemented communities (S2 Table). We calculated community phenotype index (CPI) for each herb-supplemented community to represent the overall capability for utilization of each sugar in each community (S2A and S2B Fig). CPI for glucose was elevated 1.5-2-fold in all studied communities (including the glucose-supplemented control culture). All sugar-specific CPI values were significantly elevated in communities supplemented with Frankincense, Kapikacchu and Shankhapushpi. The response to other medicinal herbs was more differentiated.

We examined taxa induced by herbal medicines in the context of their sugar utilization capacity. The average number of relevant sugar utilization pathways encoded by taxa induced by most herbs (8–10) was greater than taxa responding to 7 or fewer herbs (Fig 2B). While the trend of saccharolytic potential is evident, we observed high variability in saccharolytic potential (absent to fully present) of taxa comprising each herb responsiveness group (0 to 10) and shown (S3 Table). These results indicate that while sugar utilization potential correlates with high medicinal herb responsiveness, many taxa encode limited or no relevant saccharolytic potential, indicating metabolism of alternative substrates.

### Glycosyl Hydrolase representation is expanded by nervine medicinal herbs

The complexity of dietary glycans consumed in human diets is mirrored by the large repertoire of glycosyl hydrolase (GH) specificities encoded in gut microbiomes. Among the microbial taxa profiled, GH family assignments from the CAZy database [37, 38] were available for 63 reference genomes. The relative abundance change of taxa encoding large repertoires of GH families was further analyzed (S4 Table). Among taxa encoding extensive GH functions, we noted that many displayed high herb responsiveness including multiple *Bacteroides* spp., *B*. *dorei*, *B*. *faecis*, *B*. *sartorii*, *B*. *thetaiotaomicron* and *B*. *vulgatus*. The relative abundance of several *Parabacteroides* spp. was also strongly increased by most of the medicinal herbs.

We multiplied the relative abundance of taxa by the number of genes in each family to determine whether medicinal herbs induced preferential selection of particular GH families (Fig 2C). Frankincense induced the largest increase in relative abundance of GHs, whereas Jatamansi displayed the smallest influence on GH representation. All other herbal medicines increased a larger number of GH families. The positive selection of GHs by medicinal herbs strongly suggests that glycan catabolism specificity is restructured and an important aspect of the microbiota modulatory capacity of each herbal medicine examined. We were unable to correlate the annotated specificities of altered GH family representation to the sugar composition of individual medicinal herbs (not shown). These results suggest that factors other than sugar composition such as sugar linkages, chain-length and degree of branching may represent the relevant selective force. Transcriptional analysis of GH expression patterns would allow more precise and relevant abundance measurements of GH functions.

### Nervine herbal medicines modulate butyrate and propionate producing species

The catabolism of glycans by GHs leads to increased availability of mono-, di- and oligosaccharides that are selectively transported and metabolized by diverse sugar fermenting bacteria. Sugar fermentation by gut microbes generates varying quantities of lactate, butyrate, propionate, acetate, formate, succinate and gases such as H_2_, H_2_S, CO_2_, CH_4_ and other products.

**Fig 3:**
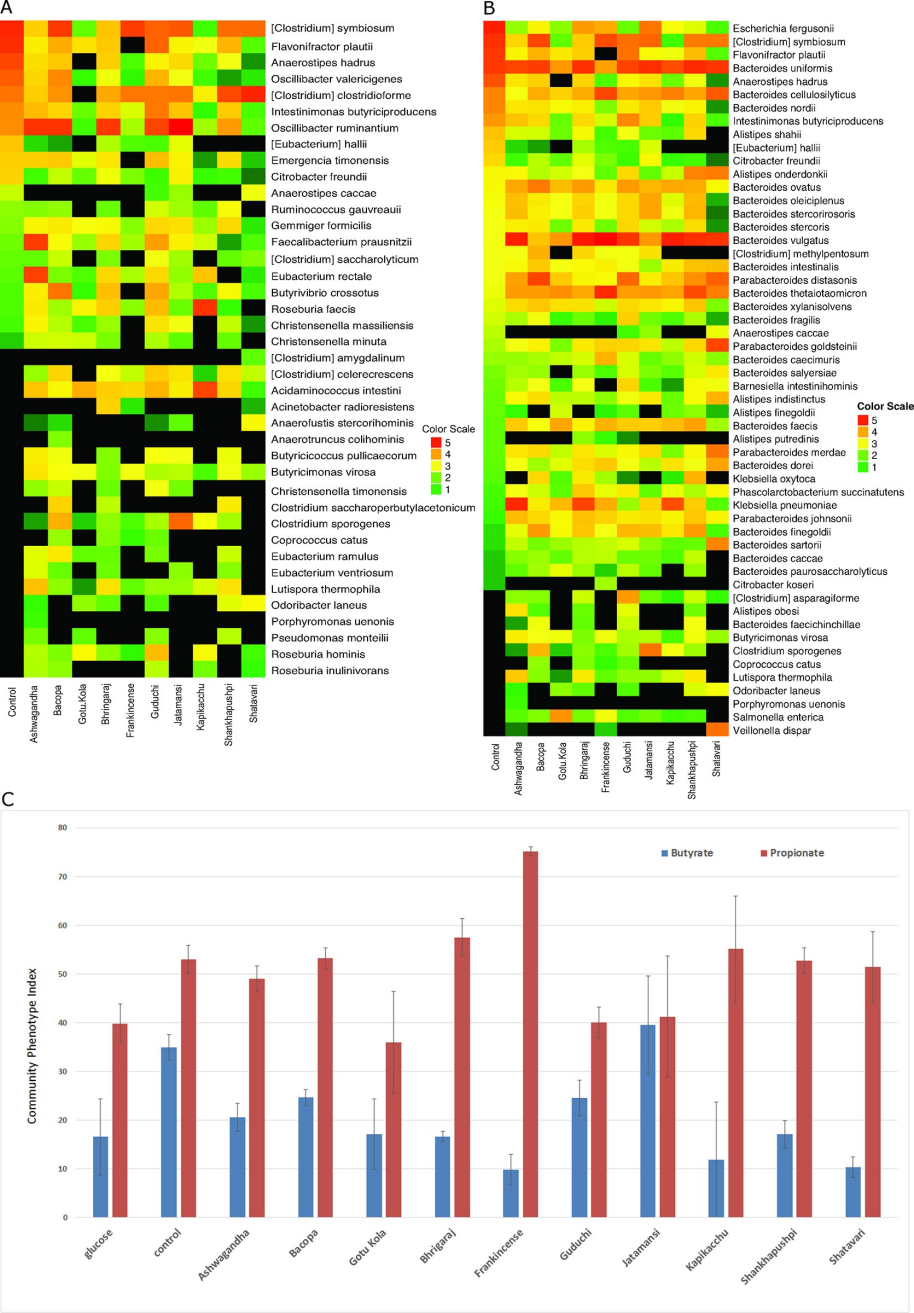
A. Herb responsiveness of predicted butyrate producing taxa. B. Predicted propionate producing taxa. Relative abundance values were multiplied by 1X10^6^ to convert all values to >1. These values were log_10_ transformed and depicted in the heatmap. C. Predicted butyrate and propionate-producing taxa. Expressed as cumulative phenotype index for each community.

We used available reference bacterial genomes to reconstruct butyrate and propionate biosynthesis pathways encoded within medicinal herb-induced communities. Four alternative pathways for butyrate production were reconstructed starting from acetyl-CoA, succinyl-CoA, L-glutamate, and L-lysine, respectively (S3A Fig). For propionate synthesis we analyzed four biochemically distinct pathway variants, namely the propanediol, acrylate, succinate pathways, and the Wood-Werkman cycle, which ferments pyruvate to propionate using the modified succinate pathway and TCA cycle (S3B Fig). These analyses resulted in the identification of a large number of taxa with SCFA biosynthetic potential, including 76 butyrate and 85 propionate producers, distributed among the medicinal herb-induced communities. We analyzed the relative abundance of these taxa in response to herb-supplementation (Fig 3A and 3B). We further calculated cumulative CPIs for butyrate and propionate in each culture condition as a prediction of relative butyrate and propionate production potential induced by each medicinal herb (Fig 3C).

Compared to control cultures, only Jatamansi selected for an increased proportion of butyrate producers. Compared to glucose-supplemented cultures, only Jatamansi, Guduchi and Bacopa selected for an increase in butyrate producers. Herbal medicine supplementation selected for distinct butyrate producers. For example, in Ashwagandha cultures, *Faecalibacterium prausnitzii* and *Eubacterium rectale* were dominant. *E*. *rectale* is the primary butyrate producer in cultures containing Gotu Kola and Kapikacchu. *Clostridium symbiosum* was dominant in response to Bacopa, Frankincense and Jatamansi and to a lesser extent Guduchi, Shankhapushpi and Shatavari. *Clostridium clostridioforme* was most dominant in cultures containing Shankhapushpi and Shatavari. Finally, *Roseburia faecis* was increased significantly by Kapikacchu supplementation.

Control cultures strongly select for propionate producers. We note that glucose-supplemented cultures selected for a single propionate producer, accounting for >99% of the total. Frankincense was the only medicinal herb that selected a greater number of propionate producers in excess of control cultures. We noted that *Bacteroides* spp. accounted for 72% of propionate-producing species in the fecal inoculum, but only 21% in control cultures. Compared to control cultures, medicinal herb supplemented cultures increased the relative abundance of predicted propionate producers. The fraction of *Bacteroides* spp. in medicinal herb-supplemented cultures ranged from 42–56% of total predicted propionate producers, with the exception of Frankincense where 77% of propionate producing species belonged to *Bacteroides*. The lack of significant alterations in SCFA producers in herb-supplemented cultures may be due to the relatively high levels of such taxa in control cultures used for comparison.

### Amino acid fermentation of herbal medicines by gut microbiota

**Fig 4:**
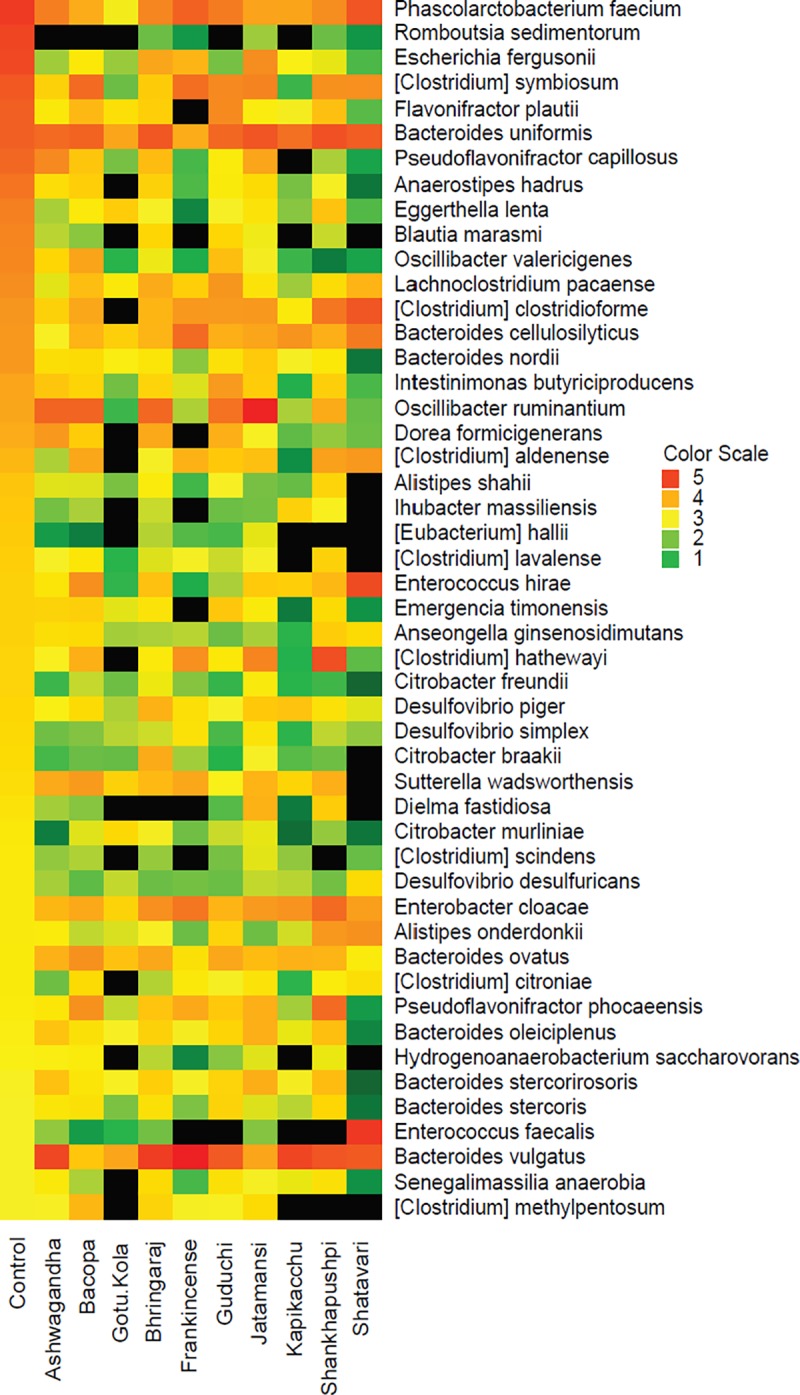
Amino acid fermenters. Taxa displaying an average relative abundance >0.1% in control cultures without carbohydrate. Relative abundance values were multiplied by 1X10^6^ to convert all values to >1. These values were log_10_ transformed and depicted in the heatmap.

We analyzed control fecal cultures (containing amino acids but no carbohydrate energy source) to identify species that engage directly in amino acid fermentation or cross feed on the products of fermentation. A total of 49 taxa displayed relative abundance in control cultures >0.1%, representing 88% of the total community (Fig 4). This community was represented by diverse taxa including; *Clostridium* spp. (8), *Alistipes* spp. (2), *Bacteroides* spp. (8), *Citrobacter* spp. (3), *Desulfovibrio* spp. (3), *Enterococcus* spp. (2), *Oscillibacter* spp. (2) and *Pseudoflavonifractor* spp. (2). The sugar utilization potential of these taxa is low (ave = 0.9 of 8 max per species). Interestingly, the relative abundance of all members of this group was increased by at least 1 medicinal herb (ave = 3.9). These results reflect the high metabolic adaptability of taxa that are both efficient amino acid fermenters and maintain high fitness in herb-supplemented cultures.

### Medicinal herb-responsiveness of asaccharolytic gut bacterial species

We identified 29 taxa that were increased in relative abundance in cultures supplemented by one or more medicinal herbs and that are predicted to encode limited or no potential to metabolize any of the sugars present in herbs (S5 Table). *Acidaminococcus intestini* and *Sutterella massiliensis* were induced by all 10 medicinal herbs. Additional taxa included *Lutispora thermophila*, *Parasutterella excrementihominis*, *Phascolarctobacterium succinatutens* were induced in 9 medicinal herb-supplemented cultures. *Blautia hydrogenotrophica* and *Eubacterium eligens* were increased by 7 herbs (S5 Table). These results may highlight taxa contributing to medicinal herb catabolism through protein catabolism and/or their amino acid fermentation capacity. A distinct set of taxa lacking relevant sugar metabolism pathways were never induced in medicinal herb-supplemented cultures including 3 *Desulfovibrio* spp., *Alistipes putredinis Eggerthella lenta*, *Emergencia timonensis*, *Flavonifractor plautii*, *Ihubacter massiliensis*, *Phascolarctobacterium faecium* and *Phocea massiliensis* (S5 Table). These taxa were highly represented in control cultures suggesting they efficiently ferment amino acids but compete less favorably when presented with medicinal herb substrates.

### Co-occurrence analysis of medicinal herb-supplemented communities

The modulatory effects of medicinal herbs act in a substrate-dependent manner and via indirect effects resulting from shifts in microbial community metabolism and the relative balance of metabolic products that cross-feed consumer species. We used 16S rRNA sequence profiles derived from 279 human fecal cultures including control cultures as well as those supplemented with 29 different prebiotics (SNP, unpublished data) and 20 Ayurvedic medicinal herbs, which included those reported here. This generated robust, statistically supported co-occurrence networks (S6 Table). We then manually inspected the co-occurring pairs of taxa, noting several pairs or larger groups of species that displayed highly similar growth profiles in one or more herb-supplemented cultures. The number of replicates for each medicinal herb was insufficient for the statistical significance of these patterns to be determined. Nevertheless, the observed patterns were so striking that we chose to analyze them in more detail. This enabled us in some cases to distinguish patterns involving phylogenetically related taxa likely based on high degree of functional redundancy (co-metabolism of substrate) or by syntrophic interactions (cross-feeding) occurring by taxonomically unrelated taxa. We exploited the observed variation in species abundance across some replicate cultures to establish additional confidence in the significance of co-varying taxa.

**Fig 5:**
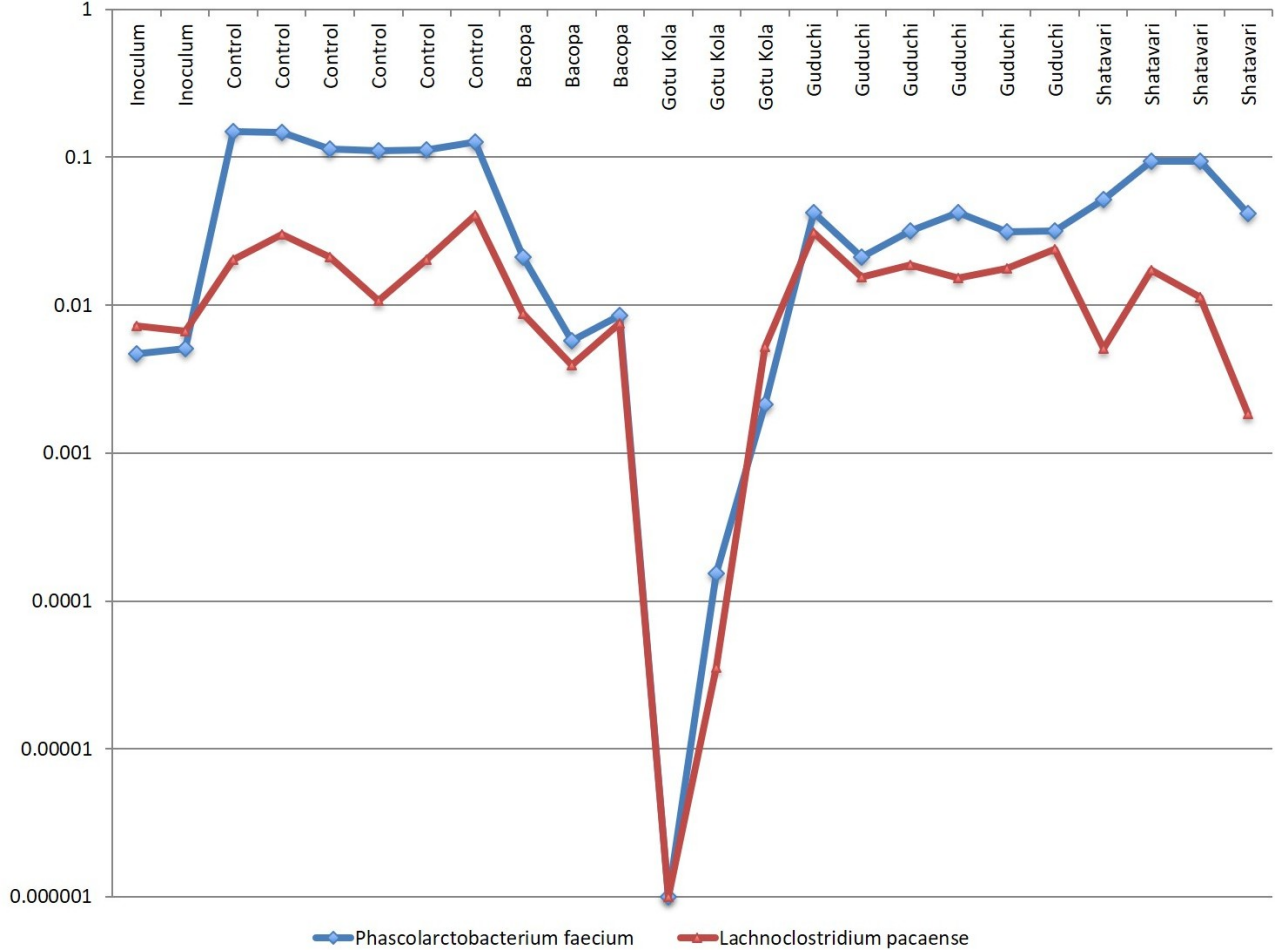
*P*. *faecium* and *L*. *pacaense* display coordinated growth. Relative abundance of taxa in replicate cultures (n = 3–6).

The relative abundance profiles of *Phascolarctobacterium faecium* and *Lachnoclostridium pacaense* bear conspicuous growth relationships in cultures supplemented with several herbs (Fig 5). These taxa were highly abundant in control cultures and generally reduced in herb-supplemented cultures. The *P*. *faecium* genome does not encode any relevant sugar metabolism pathways and utilize succinate to generate acetate and propionate. Conversely, *L*. *pacaense* encodes broad sugar utilization pathways and is predicted to generate butyrate.

**Fig 6:**
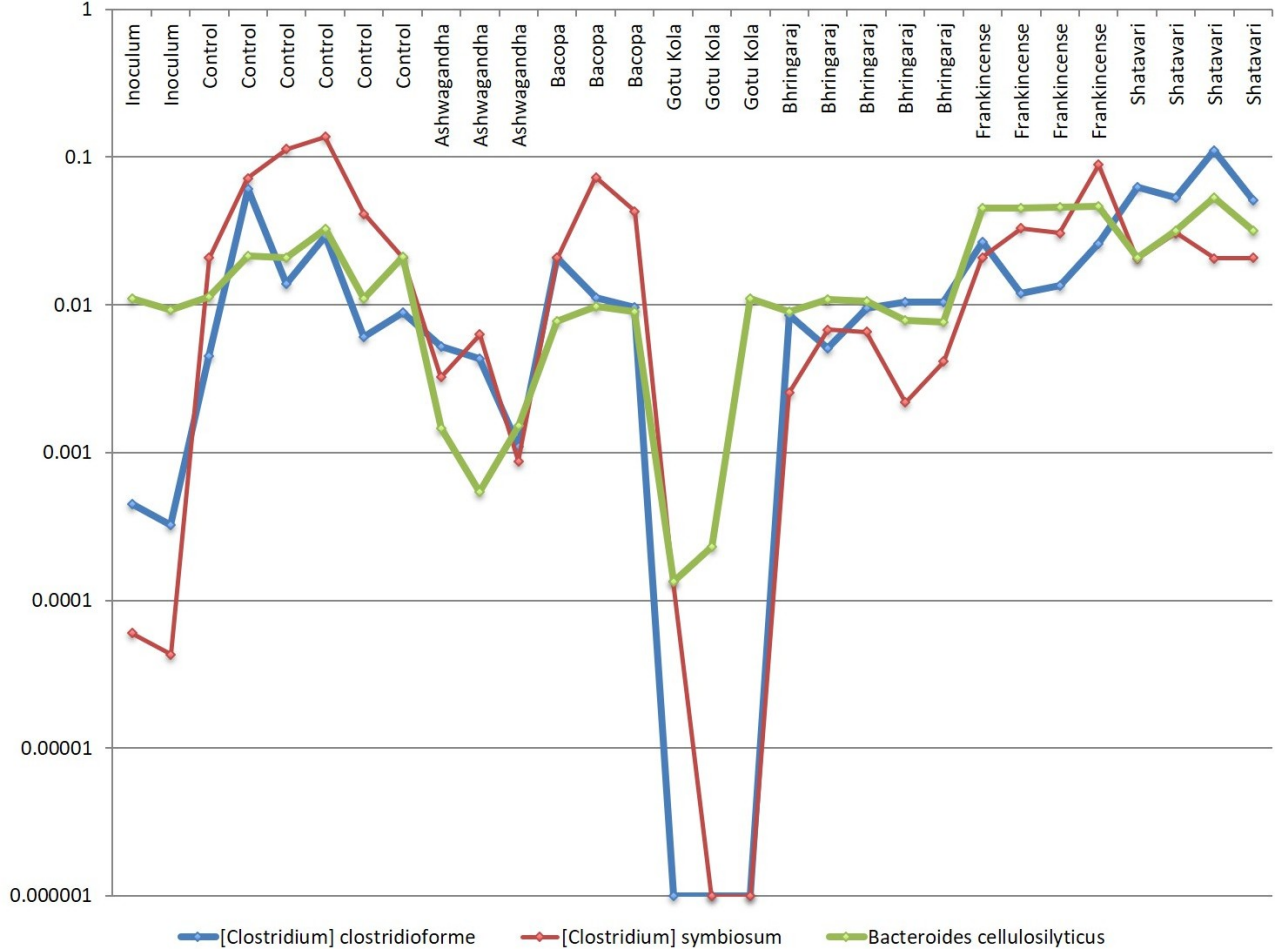
Putative consortium involving *B*. *cellulosilyticus*, *C*. *symbiosum* and *C*. *clostridioforme* display coordinated growth. Relative abundance of taxa in replicate cultures (n = 3–6). *C*.*c*. and *B*.*c*. in control, Bacopa, Bhringaraj and Shatavari. *C*.*c*. and *C*.*s*. in Ashwagandha, Gotu Kola, *C*.*s*. and *B*.*c*. in Bacopa.

*C*. *clostridioforme*, *C*. *symbiosum* and *Bacteroides cellulosilyticus* display coherent growth relationships in cultures containing multiple herbs (Fig 6). These taxa are efficient amino acid fermenters based on their high abundance in control cultures, but also encode broad sugar utilization pathways. The relative abundance of all of these species is unchanged or reduced in all medicinal herb-supplemented cultures.

Additional similarities in growth profiles were noted between pairs or groups of species. Analysis of these relationships revealed that co-varying taxa do so in a substrate-dependent manner. Moreover, it appears that some species possess elevated capacity to engage in cooperative metabolism as evidenced by the large number of distinct taxa with shared growth characteristics. Finally, we note that some medicinal herbs induce a larger number of co-varying taxa than others.

**Fig 7:**
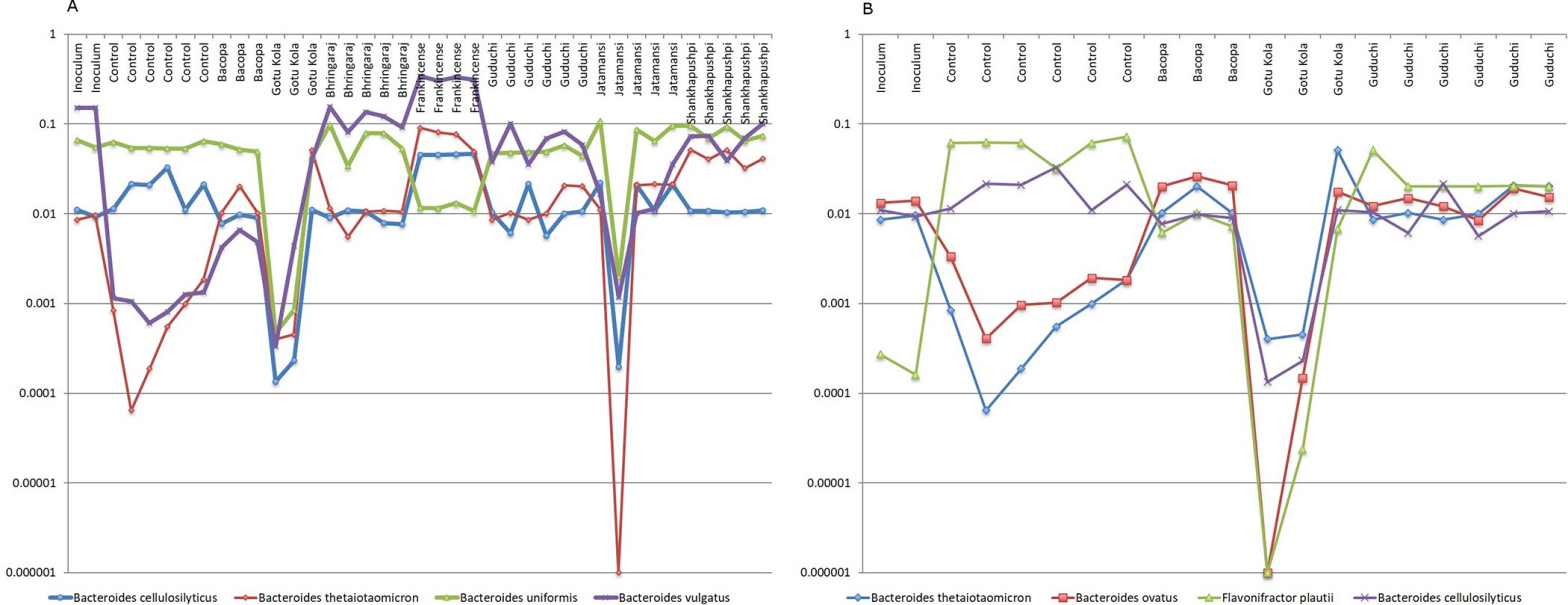
A. Putative *Bacteroides* consortium. Relative abundance of taxa in replicate cultures (n = 3–6). Bacopa, *B*.*c*., *B*.*t*. and *B*.*v*. Gotu Kola, *B*.*c*., *B*.*t*. *B*.*u*. and *B*.*v*, Bhringaraj, *B*.*t*., *B*.*u* and *B*.*v*., Frankincense, *B*.*u*. and *B*.*v*. Guduchi, *B*.*t*. and *B*.*v*. Jatamansi, *B*.*c*., *B*.*t*. *B*.*u*. and *B*.*v*. Shankhapushpi, *B*.*t*. and *B*.*u*. B. control, *B*.*t*. and *B*.*o*., Bacopa and Gotu Kola, *B*.*t*., *B*.*o*., *F*.*p*. and *B*.*c*. Guduchi *B*.*t*. and *B*.*o*.

A putative consortium including; *Bacteroides cellulosilyticus*, *B*. *thetaiotaomicron*, *B*. *uniformis* and *B*. *vulgatus* display striking growth pattern similarities that highlight the herb-dependency of these putative interactions (Fig 7A). *B*. *thetaiotaomicron* and *B*. *vulgatus* displayed increased relative abundance compared to controls in all medicinal herb-supplemented cultures, whereas *Bacteroides cellulosilyticus* and *B*. *uniformis* were abundant in control cultures (2% and 5.7% respectively) and remained largely unchanged by medicinal herb supplementation. The profiles of *B*. *thetaiotaomicron* and *B*. *uniformis* are strikingly similar in replicate cultures for some herbal medicines but not others. Replicate cultures supplemented with Jatamansi and Gotu Kola displayed high variation that serve to reinforce the potential significance of the observed growth pattern relationships of these taxa.

*B*. *thetaiotaomicron* and *Bacteroides cellulosilyticus* also display growth patterns similar to *B*. *ovatus* and *Flavonifractor plautii* (Fig 7B). Again, we note that the pair-wise combinations of taxa are medicinal herb-dependent. *B*. *ovatus* encodes sugar utilization pathways for all 8 monosaccharides present in the medicinal herbs and displays slight increases in relative abundance in several medicinal herb-supplemented cultures, whereas *F*. *plautii* encodes none of the relevant sugar utilization pathways and displays reduced relative abundance in medicinal herb-supplemented cultures. The *Bacteroides* spp. are propionate producers, whereas *F*. *plautii* generates butyrate from lysine metabolism.

**Fig 8:**
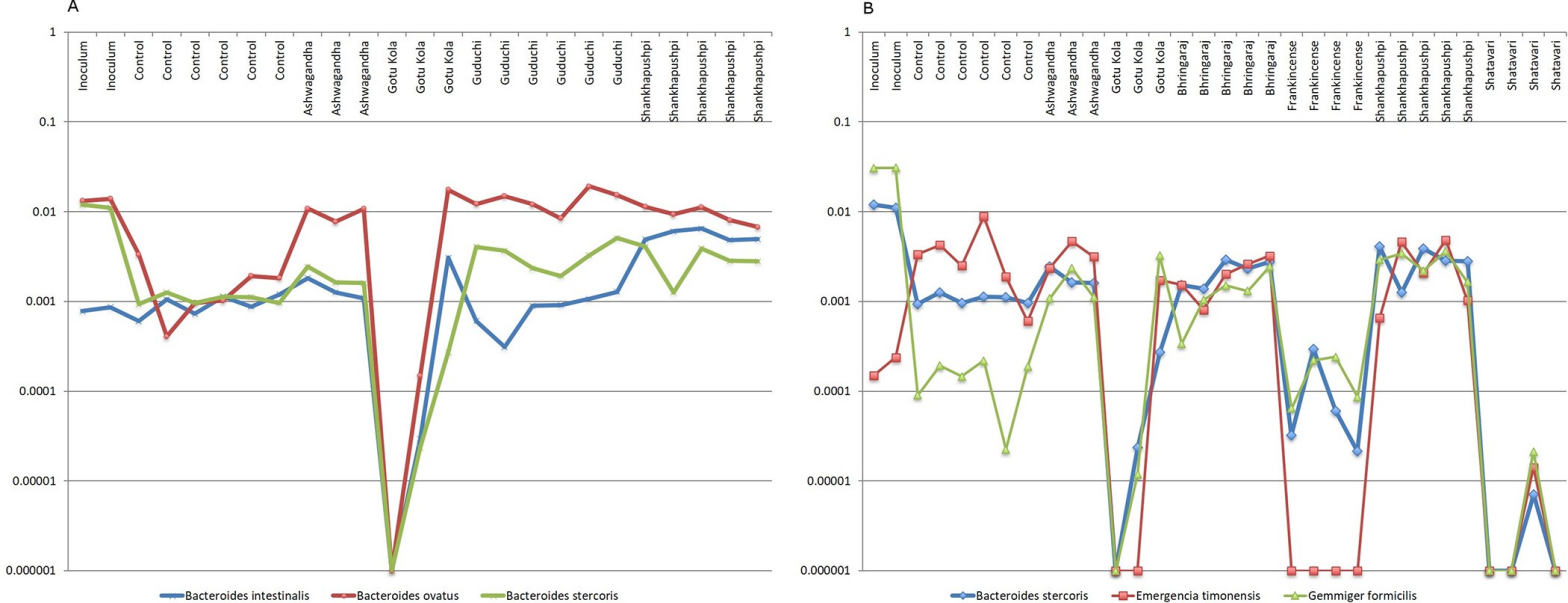
A. Putative *Bacteroides* consortium. Relative abundance of taxa in replicate cultures (n = 3–6). Ashwagandha, Gotu Kola and Shankhapushpi, *B*.*i*. *B*.*o*. and *B*.*s*. Guduchi, *B*.*o*. and *B*.*s*. B. Ashwagandha, *E*.*t*. *and G*.*f*., Gotu Kola, Frankincense, Shankhapushpi and Shatavari *B*.*s*., *E*.*t*. and *G*.*f*., Bhringaraj, *B*.*s*. and *E*.*t*.

The growth of taxa including; *B*. *ovatus* was also similar to *B*. *intestinalis* and *B*. *stercoris* display herb-dependent similarities (Fig 8A). The growth of *B*. *stercoris* was in turn related to the butyrate-producing species, *Emergencia timonensis* and *Gemmiger formicilis* in cultures supplemented with six different herbs (Fig 8B). *E*. *timonensis* does not encode relevant sugar metabolism pathways whereas *G*. *formicilis* encodes 4 of 8 relevant pathways.

The growth of *Bacteroides oleiciplenus* displayed highly similar growth patterns to *Intestinimonas butyriciproducens* in several herb-supplemented cultures and in cultures containing Ashwagandha, Bacopa, and Shankhapushpi; their growth patterns were similar to *Methanobrevibacter smithii and G*. *formicilis*. The relative abundance of *M*. *smithii* is low in control cultures, but increased substantially in several medicinal herb-supplemented cultures, suggesting that in some herbal medicine cultures, *M*. *smithii* consumes the products of metabolism, acetate and H_2_ to produce methane. This putative consortium highlights a potential cross-feeding relationship among sugar metabolizing taxa and a methanogen. *Bacteroides nordii* displayed growth similarities to *M*. *smithii* in cultures supplemented with Ashwagandha, Bacopa, Bhringaraj and Jatamansi whereas *Alistipes indistinctus* displayed patterns similar to and *B*. *oleiciplenus* (S4 Fig).

Finally, a strongly linked growth pattern observed between *Desulfovibrio piger*, an asaccharolytic H_2_ consumer, and *Bacteroides ovatus* was evident in cultures supplemented with Bhringaraj, Frankincense and Jatamansi (S5A Fig). *Bacteroides intestinalis* is also part of this consortium displaying divergent growth profile similarities to other member species in an herb-specific manner (S5B Fig).

**Fig 9:**
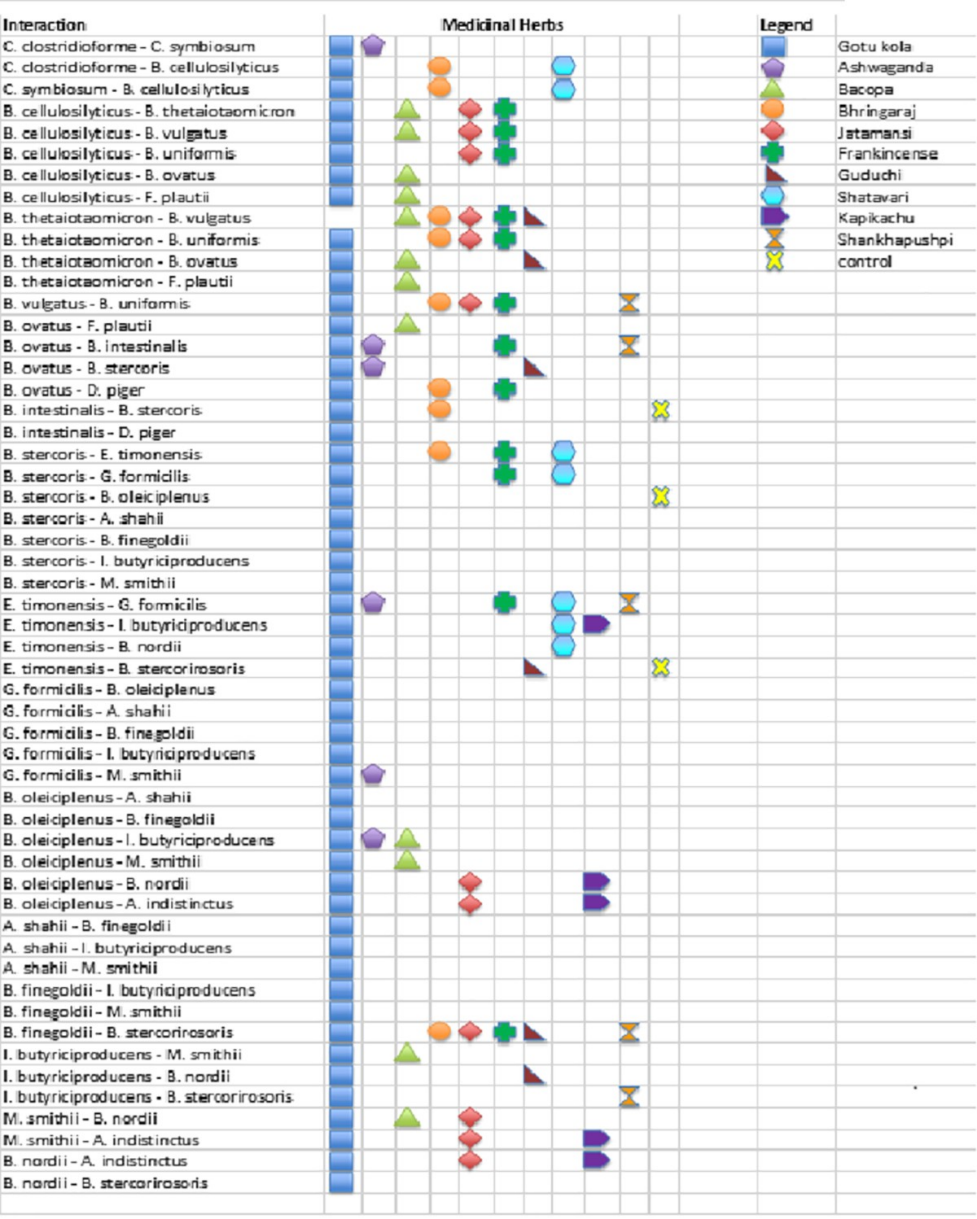
Herb-specific taxa pairs. Summary of taxa displaying growth pattern similarities in replicate cultures.

Additional putative consortia identified are summarized in (Fig 9**)** and suggest that some taxa are more frequently involved in unique herb-microbe interactions than others. These species may provide an important functional capacity to gut communities that combines broad catabolic specificity with high levels of cooperative metabolism. Additional studies are required to validate the observed growth pattern relationships and the functional basis for their presumed interactions.

## Discussion

The application of anaerobic fecal cultivation provides a means of characterizing the impact of medicinal herbs on gut microbiota composition in the absence of complicating host influence. This simplification allowed us to gain insights to the functional interactions and cooperative catabolism and metabolism of gut communities in the context of nervine medicinal herbs. Herbal medicines are a unique prebiotic, as they provide diverse glycan and protein substrates that gut microbes are well equipped to utilize. We analyzed the relative abundance patterns of taxa in medicinal herb-supplemented cultures focused on: 1. GH-mediated degradation of complex carbohydrate, 2. sugar and 3. amino acid fermentation, and 4. possible cross-feeding relationships that form in response to these processes.

### Nervine medicinal herbs alter fecal microbiota

Each medicinal herb promoted unique and strong alterations in microbial communities compared to control fecal cultures that provide amino acids but no carbohydrate energy source. PERMANOVA analysis (Bray Curtis) was used to identify the significance of the differences in community composition observed. All herb cultures were significantly different than control cultures (p<0.01–0.002). Each herb-supplemented culture was significantly different than all others (p<0.04–0.002), with three exceptions. Communities generated in Ashwagandha, Bacopa and Gotu Kola were not significantly different with respect to each other. Shatavari, Jatamansi, Kapikacchu and Guduchi drive more discrete communities compared to the other herbal medicines (Fig 1A), and within-replicate cultures generated highly distinct communities in Ashwagandha, Bacopa, Gotu Kola, Frankincense, Kapikacchu and Shankhapushpi distinct from control cultures. The occurrence of “alternative communities” in replicate cultures suggests that multiple microbial configurations may compete for substrate utilization with rather similar efficiency. The relative abundance of 103 taxa was increased by most herbs (≥5), thus highlighting community members with functionally diverse catabolic capacity (S3 Table). Distinct patterns in community representation were evident at the family-level (Fig 1C), suggesting that induced taxa encode and employ redundant or complimentary gene functions in response to medicinal herb-specific substrates. Among the ten herbal medicines evaluated in this study, each altered a large number of taxa in an herb-specific manner, thus highlighting the potent modulatory potential of medicinal herb substrates (Fig 1B).

### Genome reconstruction of sugar metabolism pathways

The reconstruction of sugar utilization phenotypes allowed us to address herb-induced taxa modulation in the context of sugar metabolism potential (S3 Table). The cumulative abundance of individual sugar metabolism pathways was altered in medicinal herb-supplemented communities (S2A and S2B Fig) but did not correlate with the sugar abundance or composition of any herbal medicine. This is consistent with our current understanding of the importance of higher order polysaccharide characteristics such as sugar linkages, chain-length and branching. We speculate a more complex modulatory capacity of medicinal herbs beyond sugar fermentation to potentially include indirect prebiotic effects from bioactive compounds and other plant components. However, we noted a relationship between the taxa displaying increased relative abundance for most herbs (7 to 10) encoded a larger compliment of sugar metabolism pathways compared to those positively responding to 6 or fewer medicinal herbs (Fig 2B). These results suggest that taxa encoding broad sugar utilization potential are more likely to display increased fitness in response to a broad array of herbal medicines and that sugar substrates present in each medicinal herb are component drivers of community composition.

Consistent with this conclusion, medicinal herb-supplemented cultures drove alterations in GH family representation and abundance in an herb-specific manner (Fig 2C). Gut microbiomes encode vast repertoires of GH specificities. The average relative abundance of several Bacteroides spp., including *B*. *dorei*, *B*. *faecis*, *B*. *sartorii*, *B*. *thetaiotaomicron*, *B*. *vulgatus*, and *Parabacteroides* spp. encode large repertoires of GHs. The relative abundance of these taxa was increased by all herbal medicines examined. Future transcriptomic studies examining GH expression patterns and sugar linkages present in each herb are expected to clarify specific substrate features of medicinal herbs driving shifts in community composition.

### Nervine herbs alter butyrate and propionate producing species

Among the most abundant taxa in control cultures (Fig 1E), nine are predicted to produce butyrate from amino acid L-lysine or L-glutamate. Similarly, among the 19 most abundant butyrate producers in control cultures (32% of the community) all but three, namely *Oscillibacter ruminantium*, *Anaerotruncus colihominis* and *Pseudoflavonifractor phocaeensis*, displayed reduced relative abundance in all medicinal herb-supplemented cultures (Fig 3A). This observation indicates that media containing only amino acid energy source select for dominant butyrate producers and that medicinal herb-supplementation results in the reduced relative abundance of these taxa. The same finding was evident for propionate producers. Among the 17 most abundant propionate producers in control cultures (57% of total), none displayed increased relative abundance in any medicinal herb-supplemented culture (Fig 3B). These results suggest that the butyrate and propionate generated in medicinal herb-supplemented cultures is primarily due to sugar fermentation. In addition, compared to control cultures, only Jatamansi selected for an increased number of butyrate producing species. Compared to glucose only supplemented cultures, Jatamansi, Bacopa, and Guduchi promoted increased numbers of butyrate producing species. These results may underplay the butyrogenic effect of medicinal herbs since they are compared to control cultures that strongly select for distinct and dominant butyrate producers.

### Amino acid fermentation

The relative abundance of 45 taxa in control culture communities (Fig 1E) were never increased by any medicinal herb tested (group 1). We noted that 49 taxa (group 2) displaying the highest relative abundance in control cultures (Fig 4) have no overlap with taxa in group-1. We deduce that taxa in group-1 are actively repressed or outcompeted in medicinal herb-supplemented cultures. Herb-repressed taxa include *Clostridium* spp. (7), *Bacteroides* spp. (4), *Desulfovibrio* spp. (3), and potential pathobionts *Citrobacter* spp. (3), *Escherichia fergusonii* and *Shigella dysentariae*. Taxa in group-2 represent those that ferment amino acids or their metabolic bi-products with high efficiency. This group was enriched in *Eubacterium* spp. (4) and *Pseudomonas* spp. Taxa in group-2 uniformly encoded limited sugar metabolism pathways (ave = 0.91 out of 8/species). These observations are consistent with their increased fitness in control cultures. Given that amino acids are not limiting in cultures, it is curious that all members of group 2 display increased relative abundance in at least 1 herb (average = 3.7) supplemented culture. Indeed 13 taxa encoded none of the 8 sugar utilization pathways, suggesting that their herb-dependent increase is due to cross-feeding on products generated by herb metabolism.

### Co-occurrence analysis of herb-supplemented communities

We noted that replicate cultures, particularly for some herbs (e.g. Gotu Kola and Shankhapushpi) displayed variable outcomes in community representation. We speculate that such variation reflects alternative community configurations that form stochastically in response to a common substrate. It is intriguing to consider that multiple, semi-independent communities may possess similar efficiencies to metabolize complex substrate, suggesting that the specific subset of taxa responding to medicinal herbs may be probabilistic.

The putative consortia we report must be interpreted with caution due to the limited culture replicates analyzed. Our results highlight a relatively large group of species equipped to ferment both amino acids and sugar for energy production. The substrate preference or possible co-expression of these pathways by these dualistic taxa in cultures supplemented with herbs remains unclear. Multiple consortia involved species belonging to a common genus. The growth pattern relationships likely reflect the presence of redundant or overlapping metabolic capacity and substrate preference. We also observed related growth profiles of phylogenetically unrelated taxa that may reflect cross-feeding relationships, such as between species with high GH diversity and sugar fermenting taxa. Additionally, consortia feature asaccharolytic taxa that derive energy from amino acid fermentation with dualistic taxa and dualists with chemolithotrophic taxa. Our results indicate that medicinal herb catabolism in culture involves both amino acid and sugar fermentation within the community. A large fraction of the taxa belonging to putative consortia possessing dual phenotypes confounds clear interpretation of the basis for their observed growth relationships.

A number of metabolites generated by gut microbes are known to support cross-feeding such as lactate, butyrate, propionate, acetate, formate, H_2_ and others. While these metabolites represent the most obvious basis for membership in proposed consortia, it is perhaps unlikely that this is the case. Virtually all of the growth relationships we observed involved pairs or trios of taxa, whereas if the SCFAs and other common metabolites were the drivers of coordinated growth, we anticipate that larger groups of taxa would be involved since numerous taxa produce and consume these metabolites. We speculate that coordinated growth profiles of species may be based on other as yet unidentified metabolites. Analysis of low complexity communities (2–5 species) *in vitro* may allow validation of these predictions and an evaluation of the molecular basis for their functional interaction.

An intriguing alternative possibility is that some taxa have evolved functions allowing them to physically interact with specific species in the community thereby facilitating the direct sharing/exchange of metabolites. In the context of herb-supplemented culture, we consider the additional possibility that taxa proximity might also be mediated through physical interactions with medicinal herb substrate. We note that the taxa displaying similar growth profiles are enriched for mucosally-associated species such as *B*. *thetaiotaomicron*, *B*. *ovatus*, *B*. *vulgatus*, *Phascolarctobacterium*, *G*. *formicilis*, *D*. *piger* and others [39, 40]. Mucosally-associated communities are distinct and adapted to forage on the consistent supply of mucin components (e.g. glycans and protein). It is interesting to speculate that mucosal-associated species may have evolved mechanisms to preferentially interact with species to foster efficient and directed cross-feeding relationships. Such relationships would be predicted to be mutually beneficial.

Support for some of the putative consortia identified here has been published. *D*. *piger* displayed striking growth similarities with *B*. *ovatus* (S5A Fig). Fermentation results in H_2_ accumulation that inhibits bacterial NADH dehydrogenases, thereby reducing fermentation efficiency. The metabolic basis of interactions between *B*. *thetaiotaomicron* and *D*. *piger* has been reported [41]. In mice, *B*. *thetaiotaomicron* increases the fitness of *D*. *piger* by providing sulfate. The *D*. *piger* genome does not encode sulfatase functions. *D*. *piger* consumes lactate, H_2_ and formate. Propionate, a major end-product of fermentation generated by *Bacteroides* spp., were lower in the fecal microbiota of mice co-colonized with *D*. *piger*. We speculate that the observed growth pattern relationships between *D*. *piger*, *B*. *ovatus* and *B*. *intestinalis* may be based on similar interactions may link their growth profiles.

Based on microbiota transcriptomics analysis, *M*. *smithii* directs *B*. *thetaiotaomicron* to preferentially ferment dietary fructans that generate acetate and formate that is subsequently consumed by *M*. *smithii* for methanogenesis [42]. *M*. *smithii* preferentially consumes formate rather than acetate when both are available. Co-colonization of mice with *M*. *smithii* and *B*. *thetaiotaomicron* resulted in large increases in cell number of both species, indicating a mutual positive fitness increase. We observed growth similarities between *M*. *smithii* and the related taxa, *B*. *oleiciplenus* and *B*. *nordii* (S4 Fig) and speculate that similar cross-feeding may link their growth profiles.

We note that some taxa are more broadly represented in interactions with other taxa dictated by medicinal herb-substrates. This may highlight an important phenotypic quality of “metabolic cooperativity”. In the context of medicinal herb metabolism, the predominantly selected for *Bacteroides* spp., *B*. *stercoris* (9 interactions), *Intestinimonas butyriciproducens* (8), *B*. *cellulolyticus* (7), *Gemmiger formicilis* (7), *B*. *ovatus* and *B*. *nordi* (6) displayed high cooperativity compared to other taxa (Fig 9).

A limitation of 16S rRNA profiling is the inability to address functional consequences of observed changes in microbiota composition. Here we employed genomic reconstruction of select energy metabolism pathways to gain functional insights and enhance the interpretation of 16S rRNA profiling data. While not definitive, this combination of data permits significant hypotheses to be generated that may be subsequently tested and validated. The analysis of the prebiotic potential of medicinal herbs represents a first step toward documenting mechanistic aspects of how gut microbiota may contribute to the therapeutic efficacy of these nervine herbs.

The medicinal herbs analyzed here are reported to alter host signaling *via* the gut-immune-brain axis [43–49]. A growing number of studies have linked the gut microbiota as a factor in the gut-brain axis [50–55]. It is intriguing to speculate that the gut microbiota modulatory capacity of these medicinal herbs may contribute to their therapeutic effect. This may occur as the result of herb catabolism that increases the bioactivity and/or bioabsorption of medicinal herbs. The bacterial metabolites produced by herb-selected communities may also alter gut and systemic immune functions. We expect that future and ongoing human interventions evaluating these herbs and their effects on gut microbiota will differ in many respects with the data reported here; however, our data suggest that medicinal herbs are potentially potent prebiotics that modulate a number of species with the potential to alter host physiology, particularly immune function. Our findings emphasize the potential relevance of gut microbiota as a factor in the mechanism of action of medicinal herbs. Additional studies involving healthy and individuals with neurodegenerative disease that include analysis of gut microbial communities and the microbially generated metabolites that gain access to the circulatory system will be of interest to improve our understanding of the variables that may positively or negatively influence the therapeutic efficacy of nervine medicinal herbs.

## Supporting information

S1 Fig Reconstructed sugar transport and catabolic pathways in reference genomes. (TIF)

S2 Fig A. Community phenotype indices. Presence (1) or absence (0) of sugar utilization pathways multiplied by relative abundance of taxa observed in each culture condition: pathways for glucose, galactose, glucuronate and galacturonate. B. Presence (1) or absence (0) of sugar utilization pathways multiplied by relative abundance of taxa observed in each culture condition: pathways for xylose, arabinose, rhamnose and mannose.
(TIF)

S3 Fig A. Reconstructed metabolic pathways for butyrate synthesis in reference genomes. Four variants of butyrate biosynthesis (P1-P4) using pyruvate, succinate, glutamate or lysine. B. Reconstructed metabolic pathways for propionate synthesis in reference genomes. Four variants of propionate biosynthesis (P1-P4) using lactaldehyde/propanediol, lactate or acetate.
(TIF)

S4 Fig Putative consortium. Relative abundance of taxa in replicate cultures (n = 3–6). Ashwaganda, Bacopa, Gotu Kola, Jatamansi and Kapikacchu, *A.i., M.s., B.n.* and *B.ol.*. (TIF)

S5 Fig A. Putative consortium *D. piger, B. ovatus* and *B. intestinalis*. Relative abundance of taxa in replicate cultures (n = 3–6). Gotu Kola, Bhringaraj and Frankincense *D.p.* and *B.o.*. B. Bacopa and Shankhapushpi, *B.i.* and *B.o.*, Gotu Kola and Frankincense, *B.i., D.p.* and *B.o.*, Bhringaraj, *D.p. and B.o.*, Shatavari, *B.i. and D.p*..
(TIF)

S1 Table 16S rDNA profiling data and statistical significance. The significance of change in relative abundance of taxa was determined using Analysis of Composition of Microbiomes (ANCOM) methodology with a False Discover Rate adjusted p-value of 0.05 threshold for significance.
(XLSX)

S2 Table Sugar utilization pathways of bacterial taxa. The presence or absence of sugar utilization pathways was scored at the species level as 1 or 0, respectively. When corresponding reference genomes were unavailable for specific taxa genus and family level assignments were predicted. In instances where multiple genomes were available and analyzed, fractional assignments were made depending on the conservation of sugar pathways.
(XLSX)

S3 Table Taxa responsiveness to medicinal herbs. Taxa displaying increased relative abundance in all 10, 9, 8, to 0 herb-supplemented cultures are shown in descending order together with predicted sugar utilization pathways and the fold change of each taxa in herb-supplemented cultures green (increased by >5-fold), yellow (unchanged, <5-fold) and red (decreased by >5-fold).
(XLSX)

S4 Table Taxa encoding GH gene families. Taxa encoding (CAZy) or predicted to encode large number of GH are shown along with the fold change of each taxa in herb-supplemented cultures green (increased by >5-fold), yellow (unchanged, <5-fold) and red (decreased by >5-fold).
(XLSX)

S5 Table Asaccharolytic taxa. Taxa are displayed in descending order of herb-responsiveness along with predicted sugar utilization pathways and fold change of each taxa in herb-supplemented cultures green (increased by >5-fold), yellow (unchanged, <5-fold) and red (decreased by >5-fold).
(XLSX)

S6 Table Co-occurrence of taxa in prebiotic/herb cultures. Taxa co-occurring among 279 cultures are shown. n00 indicates number of cultures where neither taxa were observed, n01 indicates the number of cultures where taxa 1 is absent but taxa 2 was detected, n10 indicates the number of cultures where taxa 1 is detected but taxa 2 was absent, n11 indicates the number of cultures where taxa 1 and taxa 2 were detected.
(XLSX)
