# Supplementary figures and images for: 16S rRNA gene profiling and genome reconstruction reveal community metabolic interactions and prebiotic potential of medicinal herbs used in neurodegenerative disease and as nootropics
Source: PLoS One. 2019 Mar 19;14(3):e0213869. doi: 10.1371/journal.pone.0213869 (PMC6424447; doi:10.1371/journal.pone.0213869)

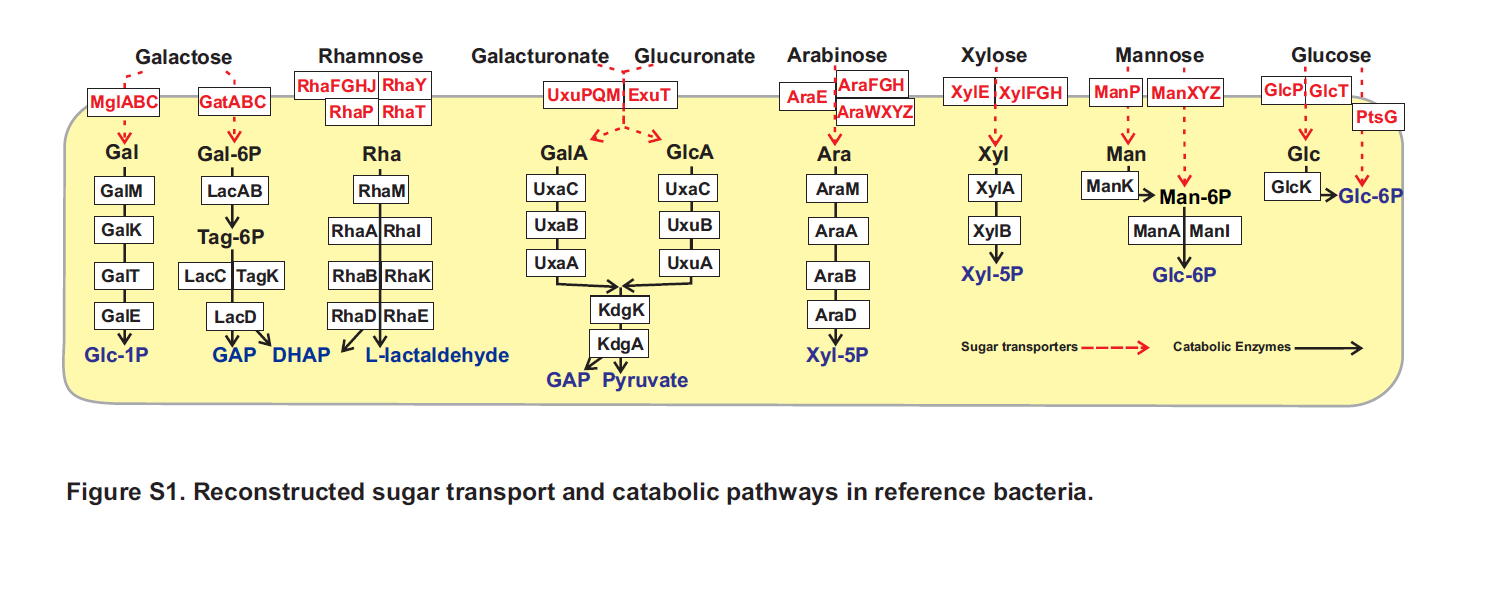

Supplement: S1 Fig — (TIF) [file pone.0213869.s001.tif]

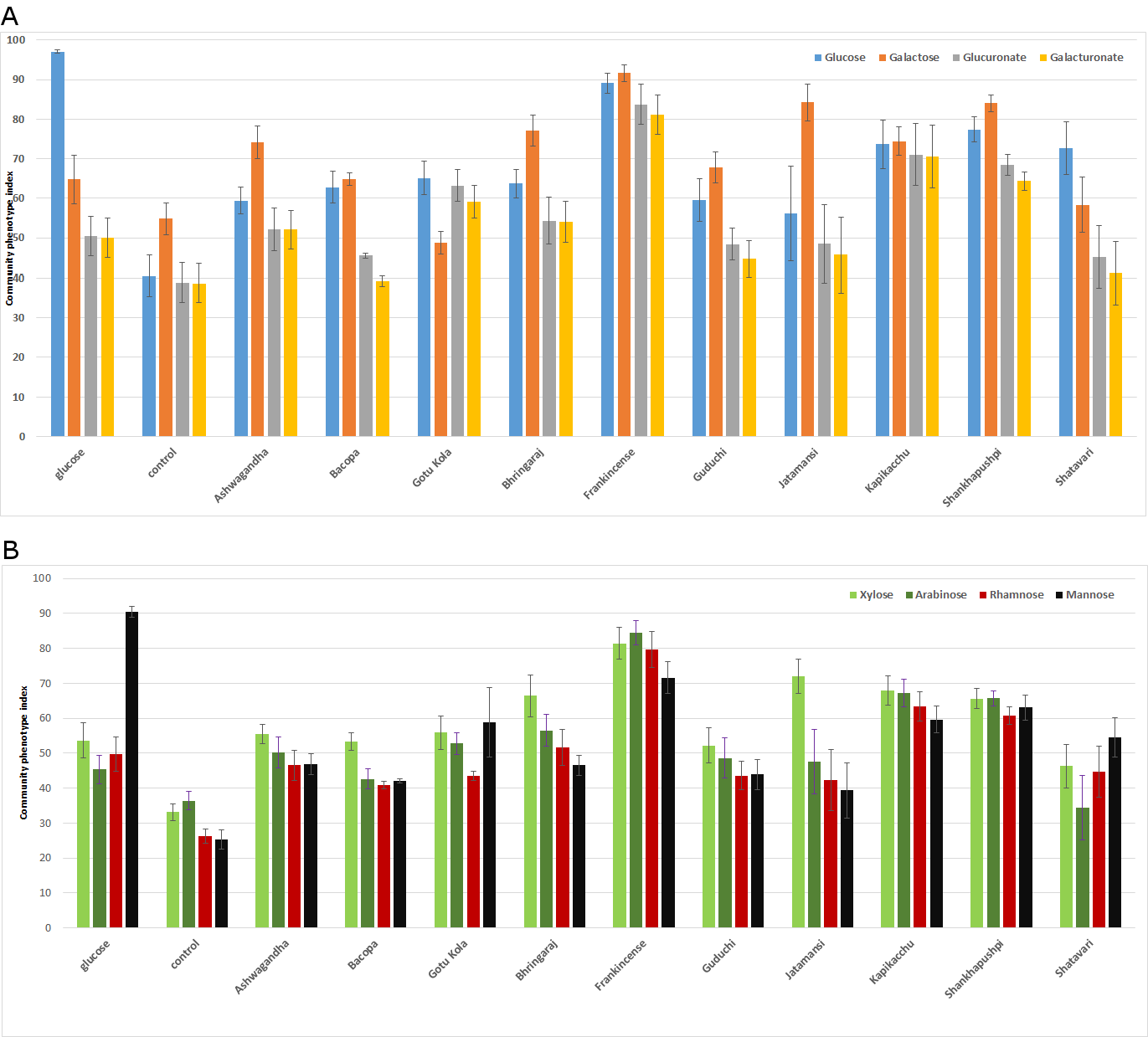

Supplement: S2 Fig — A. Community phenotype indices. Presence (1) or absence (0) of sugar utilization pathways multiplied by relative abundance of taxa observed in each culture condition: pathways for glucose, galactose, glucuronate and galacturonate. B. Presence (1) or absence (0) of sugar utilization pathways multiplied by relative abundance of taxa observed in each culture condition: pathways for xylose, arabinose, rhamnose and mannose. (TIF) [file pone.0213869.s002.tif]

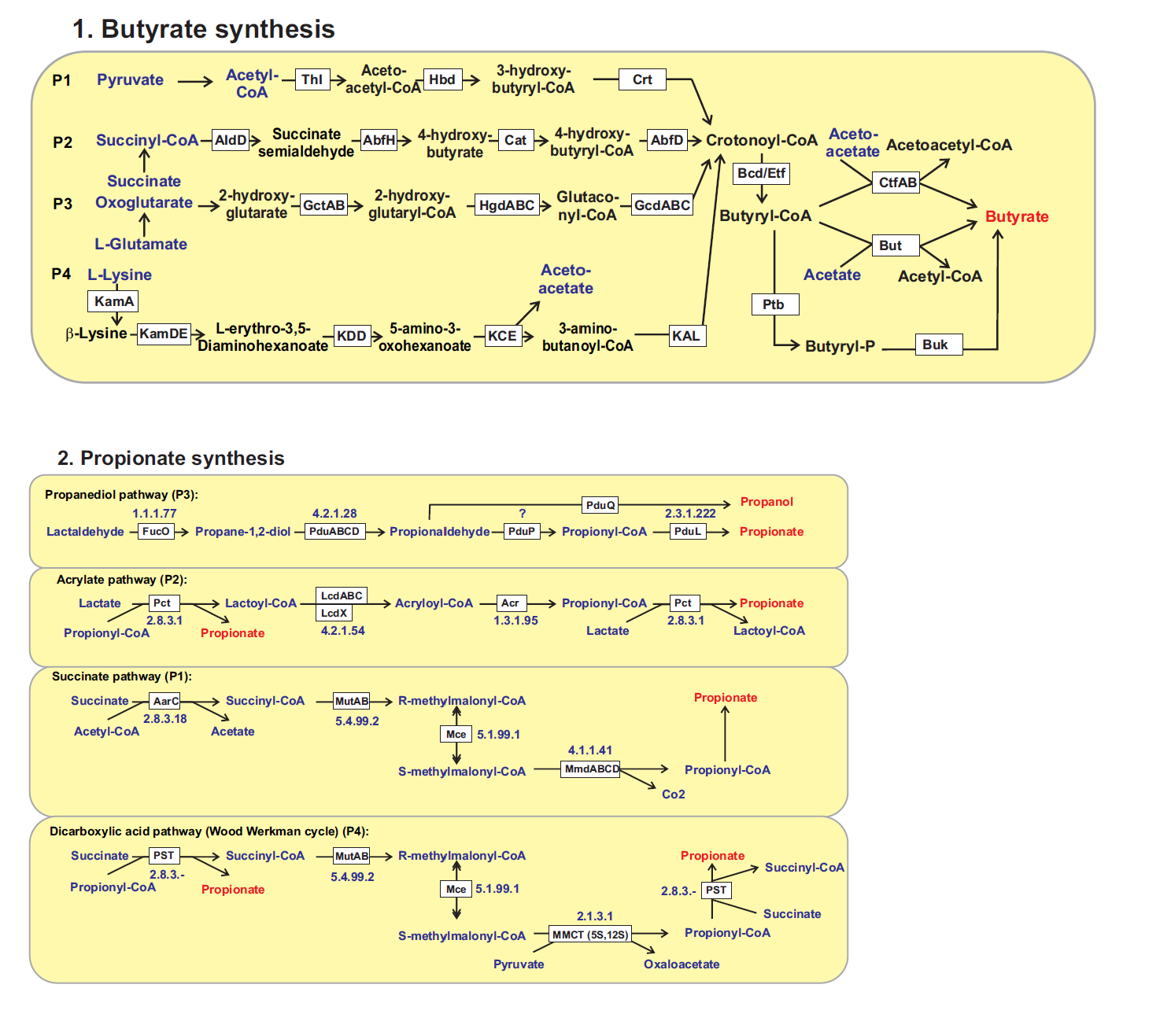

Supplement: S3 Fig — A. Reconstructed metabolic pathways for butyrate synthesis in reference genomes. Four variants of butyrate biosynthesis (P1-P4) using pyruvate, succinate, glutamate or lysine. B. Reconstructed metabolic pathways for propionate synthesis in reference genomes. Four variants of propionate biosynthesis (P1-P4) using lactaldehyde/propanediol, lactate or acetate. (TIF) [file pone.0213869.s003.tif]

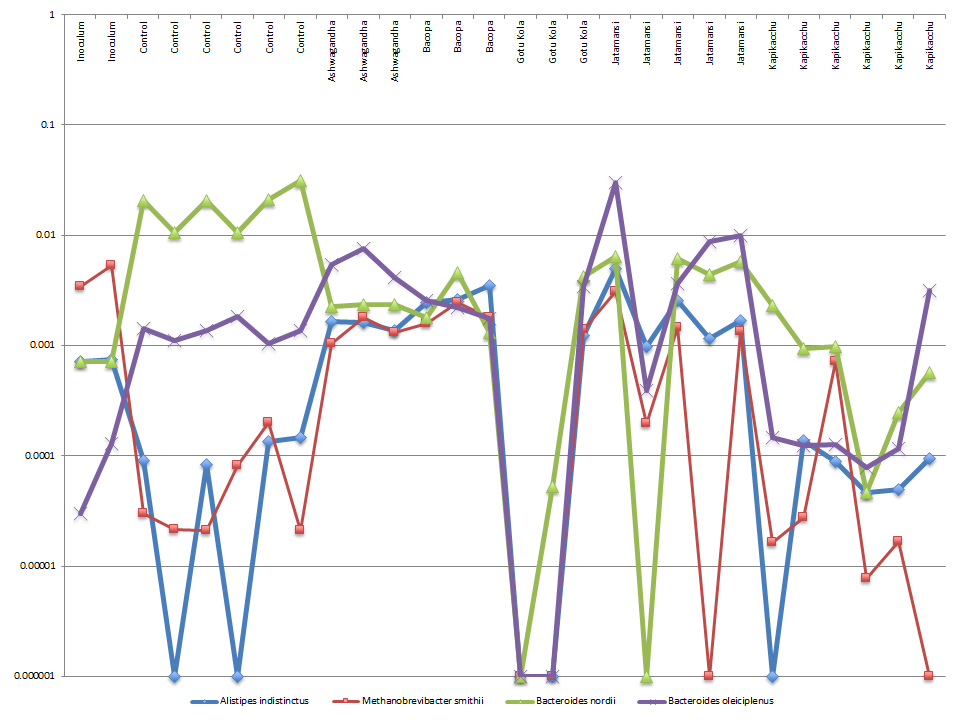

Supplement: S4 Fig — (TIF) [file pone.0213869.s004.tif]

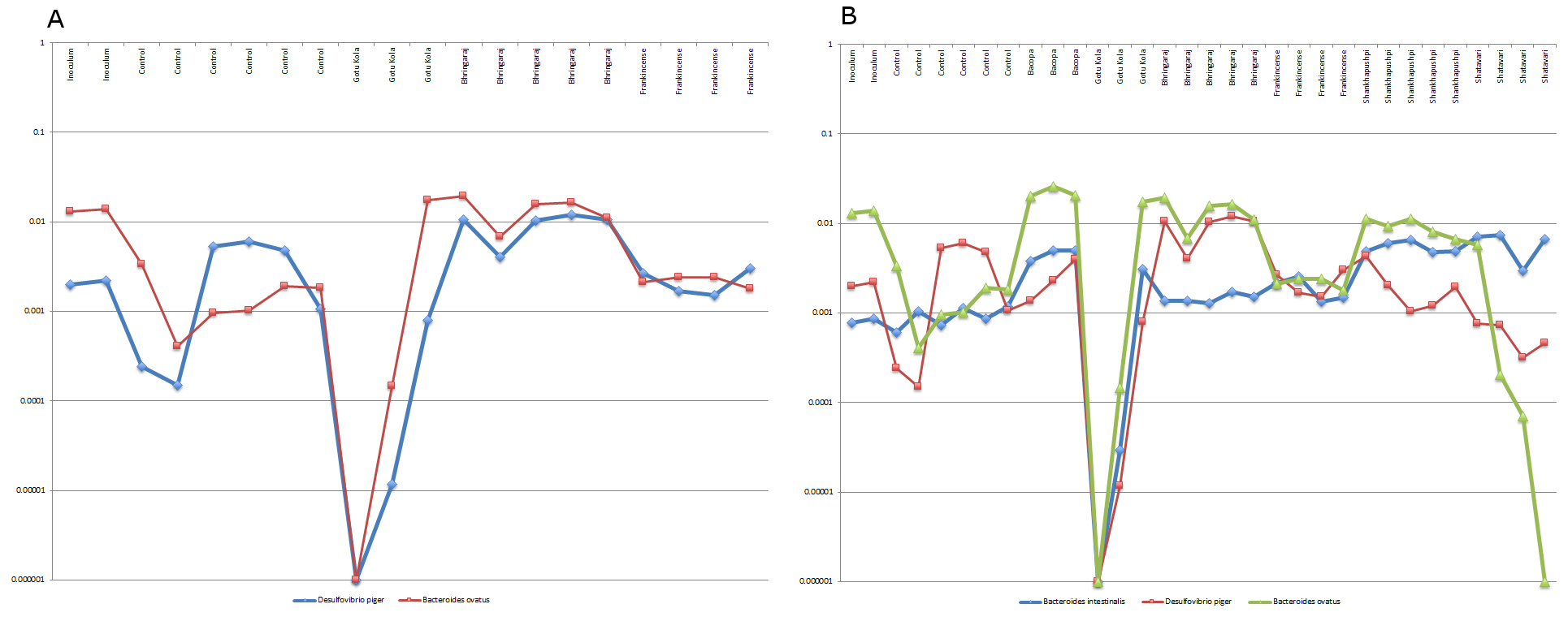

Supplement: S5 Fig — A. Putative consortium D. piger, B. ovatus and B. intestinalis. Relative abundance of taxa in replicate cultures (n = 3–6). Gotu Kola, Bhringaraj and Frankincense D.p. and B.o.. B. Bacopa and Shankhapushpi, B.i. and B.o., Gotu Kola and Frankincense, B.i., D.p. and B.o., Bhringaraj, D.p. and B.o., Shatavari, B.i. and D.p.. (TIF) [file pone.0213869.s005.tif]
